# Are Supplemental Nutrition Assistance Program Restrictions on Sugar-Sweetened Beverages Effective in Reducing Purchase or Consumption? A Systematic Review

**DOI:** 10.3390/nu16101459

**Published:** 2024-05-12

**Authors:** Charles Alba, Xi Wang, Ruopeng An

**Affiliations:** 1 Division of Computational & Data Sciences, Washington University in St Louis, St. Louis, MO 63130, USA; alba@wustl.edu; 2 Brown School, Washington University in St Louis, St. Louis, MO 63130, USA; w.xi@wustl.edu

**Keywords:** Supplemental Nutritional Assistance Program, sugar-sweetened beverage, food stamps

## Abstract

The participants in the Supplemental Nutrition Assistance Program (SNAP) consume greater amounts of sugar and sweetened beverages (SSBs) compared to non-eligible individuals, which could result in potential negative health outcomes. This can be attributed to the lack of restrictions on SSB purchases with SNAP benefits. In view of the increasing calls from advocates and policymakers to restrict the purchase of SSBs with SNAP benefits, we performed a systematic review to assess its impact towards SSB purchases and consumption. We searched articles from five databases—Cochrane, EBSCO, SCOPUS, Web of Science, and PubMed—and selected seven studies, four of which were randomized controlled trials (RCTs) and three were simulation modeling studies. All three simulation studies and one RCT reported outcomes in terms of consumption, while the other three RCTs reported outcomes in terms of purchases. All seven studies found that an SSB restriction led to a decrease in SSB consumption or purchases, with six studies reporting significant results. Nonetheless, limitations exist. These include limited studies on this subject, potential workarounds circumventing SSB restrictions, like making purchases using personal cash, potentially differed estimated effects when combined with incentives or other initiatives, and the limited geographical scope among the selected RCTs.

## 1. Introduction

The Supplemental Nutrition Assistance Program (SNAP) aims to reduce food insecurity among America’s most socio-economically vulnerable households by providing cash assistance to low-income families [1]. Previously known as food stamps, it was renamed SNAP in the 2008 Farm Bill to underscore a broader emphasis on nutrition and health to improve the overall well-being of its recipients [2]. Given that 41.2 million individuals across 21.6 million [3] low-income households rely on SNAP to combat food insecurity, the program serves as the perfect avenue to promote the preventive care benefits gained through improved dietary quality. Specifically, by encouraging SNAP recipients to consume foods with greater nutritional value, SNAP has the potential to significantly reduce the prevalence of obesity [4], type-2 diabetes [4], and cardiovascular disease [5] among low-income Americans. This could result in tremendous healthcare cost savings [6] and, in the long run, improve the quality of life for the recipients [2,6]. These improvements could be particularly beneficial for children in families receiving SNAP benefits, given that children with healthier diets tend to demonstrate better academic performance [7]. Promoting healthier diets through SNAP could therefore serve as an opportunity to elevate the economic status of these children who depend on the program to meet their nutritional needs.

Despite this focus on dietary quality, studies have demonstrated that SNAP recipients consistently fall short of meeting the recommended dietary guidelines. Singleton et al. [8] showed that SNAP participants achieve a Healthy Eating Index (HEI-2015) score that is 6.3 points lower compared to income-ineligible non-participants. This can be partly attributed to the fact that SNAP participants consume greater amounts of SSBs in comparison to non-eligible recipients [9]. Specifically, a USDA report [10] revealed that recipients spend as much as one in ten SNAP dollars on SSBs. This discrepancy can be attributed to the absence of purchase restrictions on SNAP benefits and the tax-exempt status of purchases made with SNAP benefits [2].

The absence of restrictions aimed at deterring the consumption of SSBs among SNAP participants is often viewed as a missed opportunity to address diet-related chronic diseases. These diseases disproportionately affect low-income individuals living below the poverty line, who are likely to interact with SNAP during their lifetime [11]. Implementing such restrictions could help reduce health disparities compared to their wealthier counterparts [12]. Advocates have noted that other United States Department of Agriculture (USDA)-administered nutrition programs, like the Special Supplemental Nutrition Program for Women, Infants, and Children (WIC), which have similar restrictions in place, have yielded success in achieving reduced levels of SSB consumption [13].

Nonetheless, initiating policy changes in SNAP could be challenging due to the program’s bureaucratic nature. Every five years, Congress passes the Farm Bill, which allocates funds to SNAP and initiates major policy changes within the program. States, on the other hand, are responsible for operating the program, including determining eligibility among applicants and issuing benefits via Electronic Benefit Transfer (EBT) cards. These cards can be redeemed at EBT-approved grocery stores [1].

Consequently, imposing restrictions on SSB purchases via EBT cards requires either legislative action by Congress or a USDA-approved waiver allowing individual states to enforce their own restrictions [2]. However, attempts by numerous states, including New York, Maine, and Minnesota, to obtain such waivers have consistently been denied [2]. While Congress has remained relatively silent on this issue, the recently proposed bipartisan SNAP Nutrition Security Act of 2023 by Senators Marco Rubio (R-FL) and Cory Booker (D-NJ) seeks to restrict purchases of SSBs and junk foods through SNAP benefits [14].

Many proponents favoring an SSB restriction argue that a restriction will prompt SNAP recipients to stop purchasing SSBs altogether and effectively reallocate their EBT benefits towards more nutritional alternatives [15]. However, some skeptics have argue that it may not be straightforward [16], as SNAP recipients will find work-arounds, such as purchasing SSBs with their own personal cash [17].

In light of growing support for actions aimed at restricting the purchases, this systematic review aims to examine whether restrictions on SSB purchases using SNAP benefits could achieve the desired outcome of effectively reducing SSB consumption among SNAP recipients.

## 2. Materials and Methods

### 2.1. Overview

This review was carried out in accordance with the Preferred Reporting Items for Systematic Reviews and Meta-Analyses (PRISMA) guidelines.

### 2.2. Study Selection Criteria

#### 2.2.1. Study Design

Studies that encompass experimental studies (e.g., randomized control trials [RCTs], pre-post interventions, or cross-over trials), observational studies (e.g., cross-sectional or prospective cohort studies), and simulation studies (e.g., micro-simulation or agent-based simulation modeling on existing datasets), were included in the review.

#### 2.2.2. Study Subjects

Study subjects included participants who were either enrolled in SNAP or deemed to be eligible for SNAP. These included studies which selected adults, heads of households, or children of families enrolled in or eligible for SNAP. Studies which targeted low-income participants without explicitly verifying or specifying their existing SNAP eligibility or status were excluded.

#### 2.2.3. Outcomes

The outcomes of interest were purchases or consumption of SSBs. We excluded studies that exclusively measured the success of SSB restrictions based on other health outcomes, such as obesity and type-2 diabetes, without measuring SSB purchase and consumption.

#### 2.2.4. Intervention

The intervention of interest was a total restriction of SSB purchases through SNAP benefits. Excluded studies include those analyzing the impact of purchase incentives without any food restrictions, such as incentives towards purchasing fruits and vegetables among SNAP participants, or those examining the combined effects of both restrictions and incentives rather than their distinctive effect. Additionally, studies that measured the impact of taxes on SSBs, as opposed to restrictions, or those where restrictions applied to a broader range of food items, such as packaged or junk foods, were excluded.

#### 2.2.5. Article Type

Original, empirical, and peer-reviewed journal publications were included. Editorials or commentaries were excluded.

#### 2.2.6. Time Period of Search

The search timeframe spanned from the inception of an electronic bibliographic database to 14 February 2024. However, since SNAP was initiated in 2008 as a successor to the food stamp program, we anticipated that a significant proportion of the selected studies would yield results from after 2008.

### 2.3. Search Strategies

We selected Cochrane, EBSCO, SCOPUS, Web of Science, and PubMed as our databases to conduct comprehensive searches for research articles. The list of keywords to search for SNAP included “SNAP”, “Supplemental Nutrition Assistance Program” or “Food Stamps”, as well as terms like “Food Assistance”, “Food Aid” or “Food Benefit Program” that accompanied terms associated with the United States or USDA, such as “US”, “USA”, “United States”, “United States of America”, “USDA” or “America”. To search for SSBs, key terms included “SSB”, “sugar-sweetened beverage”, “soft drink”, “fizzy drink”, “fizzy beverages”, “carbonated drink”, “carbonated beverage”, “pop” or “cola”. For EBSCO, SCOPUS, Web of Science and PubMed, searches were restricted to English publications. Additionally, for Web of Science, the search was limited to “USA”. For EBSCO, academic journals were selected as the source type, and the following directories were chosen: Exploring Race in Society, Academic Search Complete, CINAHL Plus, EconLit with Full Text, Global Health, Global Health Archive, MEDLINE, SocINDEX with Full Text, OpenDissertations, and The Serials Directory. The exact search algorithms used for each database are provided in the Supplementary Materials. C.A. and X.W. independently screened the title and abstract for articles that were found through the above-mentioned keyword search, and obtained potentially relevant articles, before reviewing their full text. The Cohen’s κ inter-rater agreement was κ = 0.657. The final set of articles was determined through a discussion between C.A. and X.W., in consultation with R.A.

### 2.4. Data Extraction and Synthesis

Through a standardized data extraction form, we collected the following information: author, publication year, and details relevant to the subjects studied. This included research’s experimental design, which included the study design, data source, study period, baseline duration, intervention duration, and statistical modeling approach (see Table 1). We also gathered information on the geographical region where participants were recruited or studied, sample size, age distribution, racial demographics, and the proportion of female participants (see Table 2). Additionally, we documented the implementation details and outcomes of each study, which included the type of restriction imposed, measures of SSB purchase/consumption, eligibility criteria of participants analyzed, and estimated effect of restrictions on SSB consumption or purchase (see Table 3).

### 2.5. Study Quality Assessment

Grading of Recommendations, Assessment, Development, and Evaluations (GRADE) was used to evaluate the quality of each selected study [13]. GRADE assigns each study one of four levels of evidence: very low, low, moderate, and high [13]. RCTs are deemed to be of high quality or evidence; whilst observational studies start at low quality or evidence due to confounding residuals [13]. The GRADE criteria enhance or diminish the quality or evidence level of a study during the evaluation process, based on factors such as risk of bias, imprecision, inconsistency, indirectness, and publication bias [14].

## 3. Results

### 3.1. Study Selection

**Figure 1:**
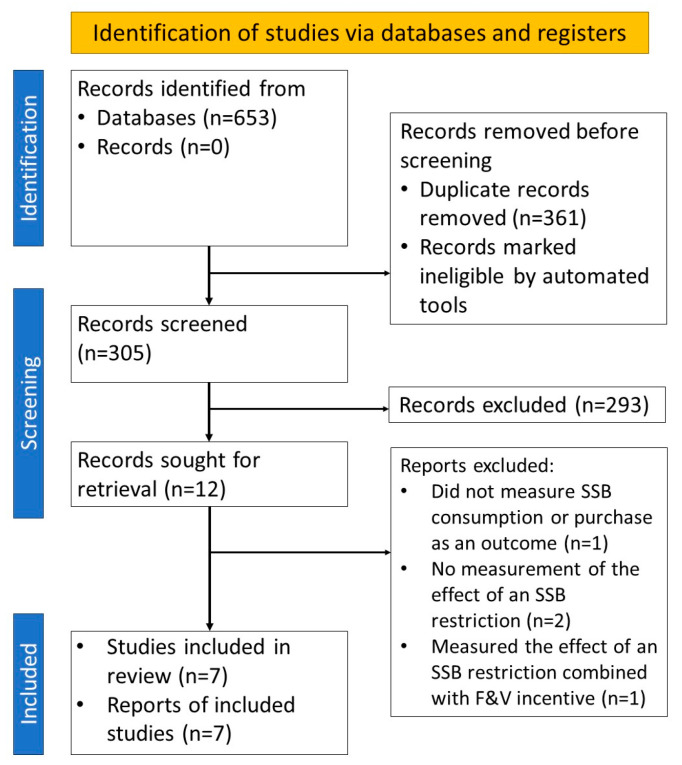
Preferred reporting items for systematic reviews and meta-analyses (PRISMA) flow diagram.

Figure 1 presents the study selection flowchart. We identified 653 articles through keyword and reference searches, including 149 from PubMed, 116 from Web of Science, 198 from Scopus, 28 from Cochrane, and 162 from EBSCO. After removing duplicates, 305 articles underwent title and abstract screening, of which 293 were excluded. The remaining 12 articles were reviewed against the selection criteria. Of these, four articles were excluded for the following reasons: one study only measured the combined effects of an SSB restriction with a fruit and vegetable incentive [23]; one did not measure SSB consumption or purchase as outcomes [24]; two did not include any SSB restriction as an intervention [9,25]; one focused on the effectiveness of a health literacy intervention [9]; and the other focused on the difference in SSB consumption between SNAP and non-SNAP participants [25]. Therefore, a final pool of seven articles [4,17,18,19,20,21,22] was included in the review.

### 3.2. Study Characteristics

Table 1 reflects the essential information for the research design for the seven selected studies, whilst Table 2 reflects details of the subjects’ characteristics for the selected studies. All studies were published within the past 10 years, with four published in the last 5 years.

#### 3.2.1. Study Design

Details for each study’s design are reflected in Table 1.

Two study designs were employed, including four RCTs [17,18,19,20] and three [4,21,22] were simulation modeling studies.

1. Modelling approaches

Among the RCTs, three studies involved monitoring purchasing behavior through receipts collected from participants during the study period [18,19,20]. All three of these studies took place in the greater metropolitan area of Minneapolis, MN. One RCT was a choice experiment, which recruited participants from across eight counties in Georgia [17].

To statistically measure the effect of interventions towards SSB consumption, Harnack et al. employed a linear regression, whilst Harnack et al. and French et al. simply calculated the outcomes in terms of group differences. Thapa et al., on the other hand, used a within-subjects ANCOVA. All methods are valid approaches in measuring the effects of an SSB restriction due to the randomized assignment of subjects in RCTs. All studies demonstrated this randomness of assignments by checking the balance and lack of difference in distribution among the covariates.

Among all the simulation modeling studies, micro-simulations were employed using variants of the National Health and Nutrition Examination Survey (NHANES) data. Specifically, Basu et al. [4] used the 1999 to 2010 data, whilst the other two used the 2009 to 2016 [21] and 2009 to 2014 [22] dataset.

2. Length of intervention

Among the RCTs, the intervention length in the non-choice experimental RCTs was either 12 or 20 weeks. Harnack et al. [18] had an intervention duration of 20 weeks (or 5 months), whilst both Harnack et al. [19] and French et al. [20] had a shorter intervention duration of 12 weeks (or 3 months). However, Harnack et al. [18] had a shorter baseline duration of 2 weeks (or 0.5 months), whilst [19] and French et al. [20] had a longer baseline duration of 4 weeks (or 1 month). This reflects the trade-off between a sufficiently long baseline duration and the length of the intervention needed to establish strong causality. Due to the nature of choice experiments, there was no baseline or intervention duration reported in Thapa’s work [17].

Among the simulation studies, all three simulation studies studied the effects of SSB restriction on SSB consumption over a hypothetical duration of 10 years [4,21,22]. This reflects the benefits of simulation studies: despite their reliance on assumptions resulting in a lack of direct evidence offered in RCTs, they can be relied upon to study the long-term effects of a potential policy.

#### 3.2.2. Subject Characteristics

Details pertaining to the subjects’ characteristics can be found in Table 2.

1. Sample sizes

Sample sizes ranged from 73 to 457 among the RCTs, and from 9753 to 139,518 for the simulation studies.

Among the RCTs, Harnack et al.’s [18] study employed a greater number of participants (*n* = 457) as their study focused on recruiting adult–child dyads, with 66 adults and 64 children being assigned to the treatment group, and 83 adults and 82 children being assigned to the control group. French et al.’s [20] study recruited a total of 256 adults, with 60 participants assigned to the treatment group and 63 to the control group. Harnack et al.’s [19] study involved a similar number of participants, recruiting a total of 265 adults, with 64 assigned to the treatment group and 66 to the control group. It is worth noting that these three RCTs also studied the effects of fruit and vegetable incentives. Both Harnack et al. [19] and French et al. [20] examined the effects of a fruit and vegetable incentive combined with an SSB restriction. Consequently, not all recruited participants were directly involved in comparisons between the control and treatment groups concerning the SSB restriction.

Thapa et al.’s [17] choice experiment had a smaller sample size of n = 73, with participants evenly assigned to four possible groups: (1) using SNAP benefits with no restrictions, (2) using cash with no restrictions, (3) using SNAP benefits with a fruit and vegetable incentive, and (4) using SNAP benefits with an SSB restriction. However, the number of participants assigned to each group was not specified.

Simulation studies, on the other hand, had larger sample sizes. Basu et al.’s [4] study included 19,388 SNAP participants and 120,130 non-SNAP participants, totaling *n* = 139,518. Both of Choi et al.’s studies exclusively examined SNAP participants, with sample sizes of *n* = 13,004 [21] and *n* = 9753 [22], respectively.

2. Subject ages

The ages of the participants varied across studies. Three of the RCTs focused solely on the adult population. Two RCTs reported a mean age of around 45 years (SD: 1.6 years) [19,20]. Meanwhile, Thapa’s [17] study involved a more senior population, averaging 54.7 years of age, although the large standard deviation of 20.7 years suggests that a wide spectrum of age groups were recruited. One study focused on adult–child dyads, resulting in a younger adult population averaging 35 years (SD: 7.5 years) and a child population averaging 7 years (SD: 2.6 years).

While ages were not explicitly stated in the simulation studies, two of the three studies explicitly focused on children below 19 years of age [21,22]. Both of these studies utilized the NHANES dataset.

3. Gender demographic

Among the RCTs, all had a disproportionately greater female population, ranging from 53% to 94.5%.

As Harnack et al.’s [18] study recruited adult–child dyads, 53% of participants were females, and 93% of the adults were females. Both Harnack et al.’s and French et al.’s studies had 81% female participants, whereas Thapa et al.’s study had the highest female demographic at 94.5%.

The disproportionately larger female demographic could be attributed to the fact that households primarily responsible for groceries were recruited for this study, which will be elaborated on in Section 3.3 below.

4. Racial Demographic

The proportion of Whites ranged from 11% to 45%, the proportion of African Americans ranged from 36% to 82.2%, the proportion of Hispanics/Latinos ranged from 3.6% to 11%, and the proportion of biracial or multiracial individuals ranged from 8% to 13.3%.

Of the four RCTs, only the study by Harnack et al. [18] had a majority Caucasian sample at 45%, with African Americans comprising 36% of the sample, and Hispanics/Latinos making up 11% of the population. This was the only one of the four RCTs to resemble the racial composition reported by SNAP, which includes 44.6% Caucasians, 27% African Americans, and 21.9% Hispanics [3]. This was also the only RCT that did not have an African American majority among the subjects.

Harnack et al.’s [19] and French et al.’s [20] studies had similar racial compositions, with 29.2% and 31.3% Caucasians, 52.7% and 51.6% African Americans, and 10% and 13.3% Hispanics/Latinos, respectively, along with 12.3% biracial individuals. Meanwhile, Thapa et al.’s [17] study recruited an overwhelming majority of African Americans, with 82.2% of recipients identifying as African American and only 11% identifying as White.

The simulation studies did not report the racial demographics.

Hence, the potential impact of an SSB restriction on SNAP benefits should be carefully interpreted, given that race is a known factor influencing SSB consumption behavior [26].

### 3.3. Eligibility Criteria of Participants

The three RCTs that monitored purchasing behavior through collected receipts recruited participants who were eligible but not presently enrolled in SNAP. The option to recruit non-enrolled participants is primarily due to legal reasons: it is not possible to alter the practices of the actual SNAP program among existing participants [19].

Harnack et al.’s [18] only eligibility criteria was that adult–child dyads be eligible but not enrolled in SNAP.

Harnack et al. [19] and French et al. [20] employed a more fine-grained criterion, specifying that participants were (1) required to have a household income of 200% of the federal poverty level or be enrolled in a government program that automatically qualifies the participant for SNAP, (2) the recruited participant who was responsible for the household’s grocery choices and purchasing and (3) could speak English, in addition to not being enrolled in SNAP for the above-mentioned legal reasons.

Because choice experiments did not alter the practices of the actual SNAP program, Thapa et al.’s [17] experiment was comprised of individuals who either participated in SNAP or qualified for SNAP, in addition to being able to speak English.

Among the simulation studies, both studies by Choi et al. [21,22] required samples to be aged below 19 years, as their study focused exclusively on children.

### 3.4. Measures of SSB Consumption or Purchases

Three studies reported outcomes in terms of purchases [17,18,20], whilst four reported outcomes in terms of consumption [4,19,21,22]. Given that there is no standardized or universal method to measure SSB consumption or purchases, each study offered distinct measurements of SSBs. Among the studies that reported outcomes in terms of purchases, two studies measured purchases in terms of average dollars spent on SSB per week (USD/wk.) [18,20] while one study measured it in terms of total purchase amounts in dollars (USD) [17]. Among the studies that reported outcomes in terms of consumption, one study measured it in terms of servings per day (servings/d) [19], one study measured it in terms of daily kilocalorie (kcal) intake per person per day [4], and two studies measured it in terms of grams per person per day [21,22].

#### Definitions of SSB

Three studies explicitly stated the definitions of SSB. Both Harnack et al. [18] and French et al. [20] defined SSBs as “water-based beverages with added sugar such as soft drinks, fruit drinks, and sports drinks”, whilst Thapa et al. [17] simply classified any sweet tea or soda drinks to encompass SSBs.

### 3.5. Types of Restrictions

Four studies defined the imposed restrictions by forbidding the purchase of sugar- sweetened beverages (SSBs) only [4,17,21,22], while three studies imposed restrictions on a combination of SSBs, sweet baked goods, and candy [18,19,20]. Although the latter is not strictly an SSB restriction, these items are closely related to each other, evident by the fact that these items are classified under the category of “Added Sugars” in the 2020–2025 Dietary Guidelines for Americans [27].

### 3.6. Estimated Effect of Restrictions on SSB Consumption

Table 3 summarizes the effects of restrictions on SSB purchases using SNAP benefits on SSB consumption and purchase. All seven studies found that an SSB restriction would lead to an overall decrease in SSB consumption.

Three out of the four RCTs reported significant differences (at *p* < 0.05) in either the purchase or the consumption of SSBs between the restricted group and the control group. Among RCTs that reported outcomes in terms of purchases, Harnack et al. [19] reported an average of USD 2.66 (SD = 0.35) spent on SSBs in the restriction group, significantly lower (at *p* < 0.0003) than the USD 4.44 (SD = 0.33) spent in the control group during follow-up. French et al. [20] also reported a reduction in average SSB spending by USD 1.40/week (SD = 0.40) in the restriction group, contrasting with an increase in SSB spending of USD +1.50/week (SD = 0.40) observed in the control group. This difference was significant (*p* < 0.05). In the choice experiment of Thapa et al. [17], participants assigned to the SSB restriction group chose significantly lower amounts of SSBs compared to groups that were given no restrictions in their use of SNAP benefits (*p* < 0.001) or cash (*p* < 0.001). Harnack et al.’s study [19] was the only RCT that reported outcomes in terms of consumption, noting an average decrease of 0.1 servings/day (SD: 0.2) in the restriction group compared to an average increase of 0.2 servings/day (SD: 0.1) in the control group during the post-intervention period compared to the baseline period. However, the difference was not statistically significant.

All three simulation studies reported that a restriction on SSBs was consistently associated with a decrease in SSB consumption, as evidenced by the 95% confidence intervals1. Specifically, Basu et al. [4], Choi et al. [21], and Choi et al. [22] reported that an SSB restriction was associated with average daily reductions in SSB consumption by 24.2 kcal/person (95% CI = 22.8, 25.5), 112.5 g/person (95% CI = 109.1, 115.9), and 108.3 g/person (95% CI = 66.0, 150.6), respectively.

All four studies that measured outcomes in terms of consumption demonstrated a reduction due to the imposed restrictions. Three of these studies reported a significant decrease. (Note: a significance level threshold of *p* < 0.05 corresponds to the 95% confidence level). However, the only RCT that reported consumption as an outcome found a non-significant decrease in SSB consumption. All three studies, which measured outcomes in terms of purchases and were all RCTs, reported a significant decrease in purchases as a result of the imposed SSB restrictions.

### 3.7. Study Quality Assessment

We assessed the quality of the studies included in the review using the GRADE framework, as reported in Table 2 [28,29]. These ratings were completed with the assistance of the GradePro GDT tool (gdt.gradepro.org Accessed on 20 April 2024) [30]. Four studies were rated as ‘High’, and three as ‘Moderate’. The three simulation studies were rated ’Moderate’ due to their large sample sizes and the robustness checks conducted in each study, such as sensitivity analysis, which enhances the precision and consistency of the studies. Nonetheless, they are still considered inferior to RCTs due to the limitations of simulation modeling, such as the lack of experimental design and the inherent reliance on assumptions in interpreting results. The four ’High’ rated studies were attributed to their adoption of an RCT design.

## 4. Discussion

### 4.1. Principal Findings

This study sought to systematically review the effects of potential SSB restrictions on their consumption and purchase among SNAP recipients. By systematically searching five databases, our review identified and synthesized findings from seven studies employing diverse methodologies. All studies found that an SSB restriction was associated with a decrease in the consumption and purchase of SSBs.

All four studies that measured outcomes in terms of consumption demonstrated a reduction due to the imposed restrictions. Three of these studies reported a significant decrease. However, the only RCT that reported consumption as an outcome found a non-significant decrease in SSB consumption. All three studies, which measured outcomes in terms of purchases and were all RCTs, reported a significant decrease in purchases as a result of the imposed SSB restrictions.

### 4.2. Implications on Health and Non-Health Outcomes amongst SNAP Participants

Decreasing SSB consumption or purchases using SNAP benefits could bring about various nutritional benefits. Among the selected studies, Harnack et al. [19] noted that 56.3% of participants assigned to the restriction group were lifted out of food insecurity or low food security compared to 31.8% in the control group. In addition, Harnack et al.’s [18] study noted no post-intervention changes in BMI z-scores amongst the restriction group, which is in contrast to the increase in BMI z-score of 0.04 witnessed amongst the control groups. Choi et al. also noted similar observations in their studies—an SSB restriction led to a 2.6 kg/m (95% CI = 2.4, 2.9) [21] and 1.2 kg/m (95% CI: 0.8, 1.5) [22] decrease in the BMIs of children. These nutritional benefits associated with excessive intake are crucial for preventing chronic diseases, with Basu et al. [4] reporting significant decreases in obesity prevalence and the incidence of type-2 diabetes of 0.89% (95% CI = 0.41%, 1.37%) and 8.5 per 100,000 population (95% CI: 2.4, 14.6), respectively. Other studies have also demonstrated distinct benefits of an SSB restriction on SNAP, such as the aversion of approximately 940,000 cardio-vascular diseases [5,23] and a gain of 2.47 million quality-adjusted life years (QALYs) [23]. In the long-run, an SSB restriction on SNAP benefits is expected to save approximately USD 41.93 billion worth of healthcare costs [23].

In addition to health improvements, initiating such a policy could have beneficial policy implications. For example, critics often use excessive SSB consumption among recipients as a scapegoat for arguing for the elimination of food stamps [31], unfairly stigmatizing well-intended recipients reliant on SNAP for food security [2]. An enforced SSB restriction, coupled with an expected shift towards healthier alternatives, could help counteract this stigma and underscore the importance of SNAP in assisting food-insecure populations. These potential benefits are parallel to SNAP’s overarching emphasis of promoting a more balanced nutritional lifestyle to achieve healthier outcomes [2].

### 4.3. Limitations

#### 4.3.1. Limited Studies

Despite performing an extensive search of five databases, only seven studies were deemed relevant to our study. This could be attributed to barriers such as the costly nature of conducting such studies, regulations that make it impossible to alter the actual SNAP program itself, and ethical concerns surrounding the need to closely monitor every single purchased item among the study’s participants. This could also perhaps explain the presence of simulation studies in our systematic review [4,21,22]. Therefore, given that 41.2 million individuals spanning 21.6 million families across all 50 states are reliant on SNAP, the limited number of studies presents challenges in generalizing the results from a limited number of areas to the entire US population.

#### 4.3.2. Limited Geographical Scope amongst RCTs

The four RCTs highlighted in our study were conducted in only two geographical regions: the greater Minneapolis (MN) metropolitan area and eight counties in Georgia. Given that certain decisions pertaining to SNAP, such as aspects of its implementation, occur at the state level, the effects of and compliance with SSB restrictions could potentially vary from state to state. Additionally, the magnitude of SSB consumption is often associated with a combination of factors like built environments [32], food desert status, and socioeconomic influences such as race [33], income [33], age demographics [34], and education levels [34].

#### 4.3.3. Work-Around from SSB Restrictions

While RCTs are known for providing high levels of evidence compared to other study designs, the intervention periods in our selected RCTs did not exceed 20 weeks (5 months). Given that the ultimate goal of the SNAP is to achieve long-term and sustainable nutritional benefits, the results reported in these studies may not fully represent the long-term effects of SSB consumption if such restrictions were in place. Participants may find work-around such as simply paying for SSB purchases using their personal cash, especially if their intentions to consume these beverages remains strong despite any restrictions.

This possibility is suggested in the qualitative findings of Thapa et al.’s [17] study, where despite quantitatively demonstrating that a restriction lead to a decrease in SSB purchases, it was noted that participants remained persistent in their desire to consume sodas despite the restrictions. They reasoned that they “love and are used to drinking sodas,” and hence would be willing to make out-of-pocket purchases towards sodas if restrictions forbid them from using SNAP benefits to make the purchase. Only a few participants acknowledged that they would halt or reduce their consumption of SSBs if they had to use their own money.

These qualitative findings could potentially suggest that SSB restrictions may be insufficient making impactful shifts in the dietary mindset of SNAP participants. The persistent desire for sodas and the willingness to purchase them with personal funds indicate that regulatory changes within SNAP might not substantially alter entrenched consumption habits.

#### 4.3.4. Differed Estimated Effects When Combined with Combined with Incentives or Educational Initiatives

This review focuses solely on the impact on restrictions towards SSB consumption or purchase. It does not consider confounding effects when paired with other policies or initiatives, such as providing incentives for fruit and vegetable consumption, educational outreach via SNAP-Ed initiatives [35], or increases in SNAP benefits to align more closely with the cost of nutritious foods [36], which are often proposed alongside an SSB restriction as part of a broader effort to reform SNAP [2]. The confounding effects resulting from pairing these initiatives could potentially reduce or negate the effect sizes observed in the reduction in SSB purchase or consumption. For instance, the study by French et al. [20] showed that pairing fruit and vegetable incentives with restrictions led to a smaller, yet still significant, reduction in SSB purchasing compared to groups with the standalone restriction.

## 5. Conclusions

This systematic review sought to investigate the effects of potential SSB restrictions on the purchase and consumption of SSBs using SNAP benefits. The main findings from all seven distinct studies reveal that restrictions on SSB purchase using SNAP benefits lead to a decrease in both the purchase and consumption of SSBs among recipients, with six of the seven studies reporting a significant estimated effect (at *p* < 0.05). Given that this reduction in SSB consumption or purchases is expected to reduce obesity [4,21,22], type-2 diabetes [4], cardio-vascular diseases [5,23], and long-term healthcare costs [23], this study reinforces the need to implement an SSB restriction as part of a broader set of reforms needed in the SNAP program. While four of the seven studies were RCTs, providing some causal evidence for a potential intervention, the limitations of our review must be acknowledged. These include limited studies on this subject, the overall limited geographical scope of the studies, the potential workaround by recipients that could render SSB restrictions unsustainable in the long run, and potentially different estimated effects when combined with incentives or educational initiatives. Hence, future RCTs related to this topic should aim to expand beyond existing studies by examining if the estimated effects remain consistent when an SSB restriction is conducted on a larger geographical scale, ideally at a state or national level, for longer periods. While this could be potentially costly to implement, the impact it could have is substantial, given the massive scale and multi-stakeholder characteristics of SNAP [2].

## Figures and Tables

**Table 1:** Detailed information of each study’s design.

Study ID	Authors (Year)	Study Design	Data Source	Study Period	Baseline Duration	Intervention Duration	Statistical Modeling Approach	Grade
1	Harnack et al. (2023) [18]	Randomized Control Trial (RCT)	Grocery Assistance Program Study (GAPS) for Families experiment participants	May of 2018 through May of 2019	2 weeks	20 weeks	Linear Regression	High
2	Harnack et al. (2016) [19]	Randomized Control Trial (RCT)	Grocery Assistance Program Study (GAPS) participants registered under clinicaltrials.gov identifier NCT02643576	August 2013 and May 2015	4 weeks	12 weeks	Group differences	High
3	French et al. (2017) [20]	Randomized Control Trial (RCT)	Grocery Assistance Program Study (GAPS) participants registered under clinicaltrials.gov identifier NCT02643576	August 2013 and May 2015	4 weeks	12 weeks	Group differences	High
4	Thapa et al. (2024) [17]	Randomized Control Trial (RCT)	Participants recruited with the assistance of Extension County Family and Consumer Sciences agents	August to December 2018	0 days (choice experiment)	0 days (choice experiment)	Within-subjects ANCOVA towards a randomized control choice experiment	High
5	Basu et al. (2014) [4]	Simulation/Modeling study	National Health and Nutrition Examination Survey (1999 to 2010)	-	-	10 years	Micro-simulation	Moderate
6	Choi, Wright, and Bleich (2021) [21]	Simulation/Modeling study	National Health and Nutrition Examination Survey (2009 to 2016)	-	-	10 years	Micro-simulation	Moderate
7	Choi, Wright, and Bleich (2020) [22]	Simulation/Modeling study	National Health and Nutrition Examination Survey (2009 to 2014)	-	-	10 years	Micro-simulation	Moderate

**Table 2:** Detailed information on the subject characteristics of each study.

Study ID	Authors (Year)	Region	Sample Size	Age Statistic	Racial Demographic	Female
1	Harnack et al. (2023) [18]	Minneapolis/St Paul (Minnesota) metropolitan area	*n* = 233 adults and *n* = 224 children, totaling to *n* = 218 families	Mean: 35 (sd: 7.5) for adults; Mean: 7.3 (sd: 2.6) for children	45% White, 36% African American, 11% Hispanic/Latino, 3% Asian-American, 1% Native American, 8% multi-racial for adults	216 (93%) for adults, 118 (53%) for children
2	Harnack et al. (2016) [19]	Minneapolis/St Paul (Minnesota) metropolitan area	*n* = 265	Mean: 44.5	29.2% White, 52.7% African American, 10% Hispanic/Latino, 13.3% biracial	214 (81%)
3	French et al. (2017) [20]	Minneapolis/St Paul (Minnesota) metropolitan area	*n* = 252	Mean: 45.0 (sd: 1.6)	31.1% White, 51.6% African American, 3.6% Hispanic/Latino, 12.3% biracial	205 (81%)
4	Thapa et al. (2024) [17]	8 counties in Georgia	*n* = 73	Mean: 54.7 years (SD: 20.7)	11% White, 82.2% African American	69 (94.5%)
5	Basu et al. (2014) [4]	United States	*n* = 19,388 SNAP participants and *n* = 120,130 non-SNAP participants	-	-	-
6	Choi, Wright, and Bleich (2021) [21]	United States	*n* = 13,004	Aged 2 to 19	-	-
7	Choi, Wright, and Bleich (2020) [22]	United States	*n* = 9753	Aged 0–19	-	-

**Table 3:** Measures and effects of SSB restrictions on SSB consumption/purchase.

Study ID	Authors (Year)	Type of Restriction	Definition of SSB	Measure of SSB Purchase/Consumption	Eligibility Criteria	Estimated Effect of Restriction on SSB Consumption/Purchase
1	Harnack et al. (2023) [18]	Not allowed to buy sugar-sweetened beverages [SSB], sweet baked goods, or candy	Water-based beverages with added sugar such as soft drinks, fruit drinks, and sports drinks	Average Dollars per week (USD/wk.)	Households, consisting of adult–child dyads, eligible for SNAP but not currently enrolled	Least-square means of USD 2.66 (sd = 0.35) on SSB in restriction group vs. USD 4.44 (sd = 0.33) in control group (*p* < 0.0003) during follow-up
2	Harnack et al. (2016) [19]	Not allowed to buy sugar-sweetened beverages [SSB], sweet baked goods, or candy	-	Servings per day (servings/d)	(1) Not currently participating in SNAP; (2) household income ≤ 200 percent of the federal poverty level or participating in a government program which automatically qualifies household for SNAP in Minnesota; and (3) adult in household most responsible for food shopping is able to read and speak English and is willing to participate.	Mean change of −0.1 servings/d (sd: 0.2) in restriction group vs. +0.2 (sd: 0.1) in control group during post-intervention period compared to baseline period
3	French et al. (2017) [20]	Not allowed to buy sugar-sweetened beverages [SSB], sweet baked goods, or candy	water-based beverages with added sugar such as soft drinks, fruit drinks, energy drinks, and sports drinks	Average Dollars per week (USD/wk.)	(1) Not currently participating in SNAP; (2) household income ≤ 200 percent of the federal poverty level or participating in a government program which automatically qualifies household for SNAP in Minnesota; and (3) adult in household most responsible for food shopping is able to read and speak English and is willing to participate.	Mean change of −USD 1.4/wk. (sd: 0.4) in restriction group vs. +USD 1.5/wk. (sd: 0.4) in control group (*p* < 0.05) during post-intervention period compared to baseline period
4	Thapa et al. (2024) [17]	Restricting SSB purchases in SNAP	Sweet tea and soda	Purchase amount in Dollars (USD)	Low-income individuals who either participate in or qualify for the SNAP program and had the ability to read and speak English	Participants assigned to the SSB restriction group choose significantly lower amount of SSBs compared to unrestricted groups with SNAP benefits (*p* < 0.001) and cash (*p* < 0.001)
5	Basu et al. (2014) [4]	Restricting SSB purchases in SNAP	-	Kilocalorie intake per person per day (kcal/person/d)	-	A net average SSB reduction of 24.2 kcal/person/d among SNAP participants (95% CI: 22.8, 25.5), which is a 15.4% decline in calorie consumption of SSBs
6	Choi, Wright, and Bleich (2021) [21]	Restricting SSB purchases in SNAP	-	Grams per person per day (g/person/d)	US children aged 2–19 years as of 2019	A reduction in SSB intake by 112.5 g/person/d (95% CI = −115.90, −109.17), approximately 4 fluid ounces on average
7	Choi, Wright, and Bleich (2020) [22]	Restricting SSB purchases in SNAP	-	Grams per person per day (g/person/d)	US children aged less than 19 years	A reduction in SSB intake by 108.3 g/person/d (95% CI: −150.6, −66.0)

## Data Availability

The search algorithms used to extract the papers from each database could be found in the Supplementary Materials.

## Supplementary Materials

The following supporting information can be downloaded at: https://www.mdpi.com/article/10.3390/nu16101459/s1.
