# Supplementary material for: Are Supplemental Nutrition Assistance Program Restrictions on Sugar-Sweetened Beverages Effective in Reducing Purchase or Consumption? A Systematic Review
Source: Nutrients. 2024 May 12;16(10):1459. doi: 10.3390/nu16101459 (PMC11123964; doi:10.3390/nu16101459)
Supplement: Supplementary file 1 [file nutrients-16-01459-s001.zip › nutrients-3003387-supplementary.pdf]

Supplemental materials for “Are SNAP Restrictions on Sugar-Sweetened Beverages Effective in Reducing Purchase or Consumption? A Systematic Review”

By Charles Alba, Xi Wang, Ruopeng An

PICOS Framework

Participants: SNAP participants or SNAP-eligible recipients
Interventions: Restriction on SSB purchases with SNAP benefits
Comparison: Restricting SSB purchases on SNAP recipients vs No restrictions
Outcomes: SSB consumption or purchases
Study Design: RCTs, observational studies (including cross-sectional, simulation modeling, etc).

Search algorithm for each database

Cochrane

```
(
(
(Sugar* NEXT (drink* OR beverage*))
(Sugar-sweetened NEXT (drink* OR beverage*))
OR (SSB*) OR "soda" OR
(can* NEXT (beverage* OR drink*)) OR
(sweet* NEXT (drink* OR beverage*)) OR
(Soft NEXT drink*)
OR (Fizzy NEXT (Beverage* OR Drink*)) OR "Pop" OR (Cola*)
OR (Carbonated NEXT (Drink* OR Beverage*)))

AND

("SNAP" OR "Supplemental Nutrition Assistance Program" OR
(("Food Assistance" OR “Food Aid” OR “Food benefit program”) AND
("US" OR "USA" OR "United States" OR "United States of America"
OR "USDA" OR "America")) OR "Food stamps"
)
):ti,ab,kw
```

EBSCO

(
AB("Sugar-sweetened beverage*" OR "SSB*" OR "soda"
OR "sweet* (drink*OR Beverage*)"
OR "Soft Drink*"
OR "Fizzy (Drink* OR Beverage*)"
OR "Pop" OR "Cola*"
OR "Carbonated (Beverage* OR Drink*)"
OR "Can* (drink* OR Beverage)"
OR "sugar* (drink* OR beverage*)")
OR
TI("Sugar-sweetened beverage*" OR "SSB*" OR "soda"
OR "sweet* (drink*OR Beverage*)"
OR "Soft Drink*"
OR "Fizzy (Drink* OR Beverage*)"
OR "Pop" OR "Cola*"
OR "Carbonated (Beverage* OR Drink*)"
OR "Can* (drink* OR Beverage)"
OR "sugar* (drink* OR beverage*)")
)
AND
(
AB("SNAP"
OR "Supplemental Nutrition Assistance Program"
OR (("Food Assistance" OR “Food Aid”) AND ("US" OR "USA" OR "United States" OR "United States of America" OR "USDA" OR "America") OR "Food stamps"))
OR
TI("SNAP"
OR "Supplemental Nutrition Assistance Program"
OR (("Food Assistance" OR “Food Aid” OR “Food Benefit Program”) AND ("US" OR "USA" OR "United States" OR "United States of America" OR "USDA" OR "America") OR "Food stamps")

)

<u>SCOPUS</u>

TITLE-ABS(
(“Sugar-sweetened beverage*”
OR “SSB*” OR “soda” OR
“sweet* (drink* OR beverage*)”

OR “Soft Drink*”
OR “Fizzy (drink* OR beverage*)” OR “Pop”
OR “Cola*”
OR “Carbonated Beverage*”
OR “Can* drink*”
OR “sugar* (drink* OR beverage*)*”
)

AND

(“SNAP” OR “Supplemental Nutrition Assistance Program” OR
((“Food Assistance” or “Food aid” OR “Food Benefit Program”) AND
(“US” OR “USA” OR “United States” OR “United States of America”
OR “USDA” OR “America”)) OR “Food stamps”)

)

Web of Science

(
AB=(“Sugar-sweetened beverage*”
OR “SSB*” OR “soda” OR
“sweet* (drink* OR beverage*)”
OR “Soft Drink*”
OR “Fizzy (drink* OR beverage*)” OR “Pop”
OR “Cola*”
OR “Carbonated Beverage*”
OR “Can* drink*”
OR “sugar* (drink* OR beverage*)*”)

OR
TI=(“Sugar-sweetened beverage*”
OR “SSB*” OR “soda” OR
“sweet* (drink* OR beverage*)”
OR “Soft Drink*”
OR “Fizzy (drink* OR beverage*)” OR “Pop”
OR “Cola*”
OR “Carbonated Beverage*”
OR “Can* drink*”

OR “sugar* (drink* OR beverage*)*”)
)

AND
(
AB=(“SNAP” OR “Supplemental Nutrition Assistance Program” OR “Food Assistance” or “Food aid” OR “Food Benefit Program”)

OR TI=(“SNAP” OR “Supplemental Nutrition Assistance Program” OR “Food Assistance” or “Food aid” OR “Food Benefit Program”)
)

PubMed

("Sugar-Sweetened Beverages" [Mesh] OR "Sugar-sweetened beverage*" [TIAB] OR "SSB" [TIAB] OR "SSBs" [TIAB] OR
"soda" [TIAB] OR
"sweet* drink*" [TIAB] OR
"sweet* beverage*" [TIAB] OR "Artificially Sweetened Beverages" [Mesh] OR
"Soft Drink*" [TIAB] OR
"Fizzy drink*" [TIAB] OR
"Fizzy beverage*" [TIAB] OR
"Pop" [TIAB] OR
"Cola*" [TIAB] OR
"Carbonated Beverage*" [TIAB] OR "Carbonated Beverages" [Mesh] OR
"Canned drink*" [TIAB] OR
"sugar* drink*" [TIAB] OR
"sugar* beverage*" [TIAB])

AND

("SNAP" [TIAB] OR "Supplemental Nutrition Assistance Program" [TIAB] OR "Food benefit program" [TIAB] OR (("Food Assistance" [Mesh] OR "Food Assistance" [TIAB] OR "Food Stamps" [TIAB] OR "Food Benefit Program" [TIAB]) AND ("United States Department of Agriculture" [Mesh] OR "United States Department of Agriculture" [TIAB] OR "United States" [Mesh] OR "United States" [TIAB] OR "United States Government Agencies" [Mesh] OR "United States Government Agencies" [TIAB] OR

"US" [TIAB] OR "USA" [TIAB] OR "United States of America" [TIAB] OR "USDA" [TIAB] OR "America" [TIAB]))
)
